# Gender disparities in high-quality research revealed by Nature Index journals

**DOI:** 10.1371/journal.pone.0189136

**Published:** 2018-01-02

**Authors:** Michael H. K. Bendels, Ruth Müller, Doerthe Brueggmann, David A. Groneberg

**Affiliations:** Division of Computational Medicine, Institute of Occupational, Social, and Environmental Medicine, Goethe University Frankfurt, Theodor-Stern-Kai 7, Frankfurt, Germany; Institut Català de Paleoecologia Humana i Evolució Social (IPHES), SPAIN

## Abstract

**Background:**

The present study aims to elucidate the state of gender equality in high-quality research by analyzing the representation of female authorships in the last decade (from 2008 to 2016).

**Methods:**

Based on the Gendermetrics platform, 293,557 research articles from 54 journals listed in the Nature Index were considered covering the categories *Life Science*, *Multidisciplinary*, *Earth & Environmental* and *Chemistry*. The core method was the combined analysis of the proportion of female authorships and the female-to-male odds ratio for first, co- and last authorships. The distribution of prestigious authorships was measured by the *Prestige Index*.

**Results:**

29.8% of all authorships and 33.1% of the first, 31.8% of the co- and 18.1% of the last authorships were held by women. The corresponding female-to-male odds ratio is 1.19 (CI: 1.18–1.20) for first, 1.35 (CI: 1.34–1.36) for co- and 0.47 (CI: 0.46–0.48) for last authorships. Women are underrepresented at prestigious authorships compared to men (Prestige Index = -0.42). The underrepresentation accentuates in highly competitive articles attracting the highest citation rates, namely, articles with many authors and articles that were published in highest-impact journals. More specifically, a large negative correlation between the 5-Year-Impact-Factor of a journal and the female representation at prestigious authorships was revealed (r(52) = -.63, P < .001). Women publish fewer articles compared to men (39.0% female authors are responsible for 29.8% of all authorships) and are underrepresented at productivity levels of more than 2 articles per author. Articles with female key authors are less frequently cited than articles with male key authors. The gender-specific differences in citation rates increase the more authors contribute to an article. Distinct differences at the journal, journal category, continent and country level were revealed. The prognosis for the next decades forecast a very slow harmonization of authorships odds between the two genders.

## Introduction

Gender inequity in science began to shift into the public eye since the 1970s driven by the movement of Second Wave Feminism, which sparked a growing interest in the subject. For almost half a century, the topic has remained in the focus of the scientific community, despite many initiatives to promote female scientists [1, 2]. Numerous publications in the recent years documented the persistence of a gender gap in science[1–4] leading to discussions about the underlying reasons[5]. Hence, the problem is omnipresent and deeply rooted in the scientific world and warrants constant re-evaluation by sound research methods.

An easy accessible and objective indicator for the successful integration of women in science is the quantification of their scholastic activity as represented by “authorship” in scientific publications [6, 7]. In this context, it is common opinion that “scientific authorship” embodies a type of reward system that does not exclusively honour the pure scientific merit of someone’s intellectual contribution but also reflects hierarchical structures of the research community [8]. In many research areas including life science, chemistry, and earth & environmental science, the position in an author list is important for reasons unrelated to the article's content, namely, prestige and eligibility for research grants. In these research areas, it is common practice that "the first author indicates the person whose work underlies the paper as a whole" [9], whereas the last authorship "indicate a person whose work or role made the study possible without necessarily doing the actual work" [9]. As a consequence, the prestige of authorships follows a ranked order with a higher reputation of first and last authorships and a lower reputation of co-authorships [7, 8, 10]. Moreover, since in the considered scientific fields, early-career researches usually publish as first or co-authors and senior researches preferably as last authors [8, 11], the analysis of authorships permits conclusion regarding the academic status of women in the hierarchical scientific system. This axiomatic view is valid for original articles; however, it should be mentioned that the order of authors is often reversed on review articles [11].

In light of this, 1.) Sugimoto and colleagues published in 2013 a global and cross-disciplinary bibliometric analysis confirming that gender imbalances persist in research output. Specifically, they showed that women are responsible for fewer than 30% of fractionalized authorships worldwide. Moreover, they demonstrated that in the most productive countries, all articles with women in key author positions receive fewer citations than those with men in the same positions [6]. 2.) In 2016, Filardo and colleagues examined changes in representation of women among first authors of original research published in high impact general medical journals from 1994 to 2014 and investigated differences between journals [12]. They reported that female first authorship increased significantly from 27% in 1994 to 37% in 2014, but seemed to have plateaued and to be in decline in some journals [12]. By applying mathematical odds ratios, they further revealed major differences in female odds to secure a first authorship between the journals. 3.) Recently, Long and colleagues [4] compared the percentage of female first and senior authors with the percentage of women practicing in academic gastroenterology. They demonstrated that the proportion of women in the senior author position is less than expected based on the proportion of women among academic gastroenterologists [4]. Specifically, they found that in 2015, 18% of the first authors, but only 10.1% of the senior authors were women. In terms of odds ratio, women had twice the odds to secure first authorships than last authorships [4].

We here applied the methodology on a big-data scale and focused on the representation of female authorships in high-quality research assessing 54 journals of the Nature Index [13]. The Nature Index was created in 2014 and offers a database for the specific analysis of global high impact scientific efforts from the journal categories of *Life Science*, *Multidisciplinary*, *Earth & Environmental*, *Chemistry* and *Physics* [13]. The group of journals was independently chosen by researchers as being where they would want to publish their most significant research [13]. The choices reflect thereby researchers' perception of the journals' content, rather than measures such as impact factor [13]. With the exclusion of the field of physics due to the lack of authors´ first names, we analyzed 1,488,989 male and female authorships from 293,557 articles that were published between January 2008 and May 2016.

The purpose of this study is to answer following main questions concerning the integration of women in high-quality research: How is the relative distribution of women among first, co- and last authorships compared to men? How is the temporal dynamics as well as the decade forecast of the female representation? Are there gender-specific differences in productivity and citation rates? Is there a tendency that the representation of women is reduced at highly competitive authorships? Specifically, what is role women tend to have in articles with many authors, e.g. collaborative projects and in articles that are published in the highest-impact journals? Finally, are there strong regional differences between countries and continents regarding the integration of women and do we reveal a correlation between the percentage of female authorships and the female representation at prestigious authorships?

To address these questions, we used odds ratios to measure the relative distribution of women among first, co- and last authorships compared to men and applied the *Prestige Index* to assess the distribution of prestigious authorships between the two genders.

## Materials and methods

### Data acquisition & integration

English original research articles were acquired from the Web of Science Core Collection (http://apps.webofknowledge.com/WOS_GeneralSearch_input.do?product=WOS&search_mode=GeneralSearch&SID=C6MGKFyzPSatF6NT7Sf&preferencesSaved=). The study period covers January 1, 2008 to May 18, 2016, yielding 293,557 articles that were published in 54 Nature Index journals [13] from the journal categories *Life Science*, *Multidisciplinary*, *Earth & Environmental* and *Chemistry*. Except for three journals (*Ecology* and *Ecology Letters* and *Nature Chemical Biology*), all journals were assigned to one single category. The data analysis was performed using the SQL-Server-based Gendermetrics.NET [14] that constitutes a further development of the NewQIS platform [15]. During data integration, authors were generated by grouping the article authorships by name and first names. This means, each author entity is associated with a non-empty set of authorships. Please note this conceptual difference between author and authorship. In total, 693,575 authors affiliated to institutions from 185 countries were considered.

### Gender determination

The algorithmic gender determination employed a data table containing the gender (male, female and unisex) of 77.818 first names, as previously described in Bendels et al. [14, 16]. In total, 313,894 (= 45.3%) male authors, 200,280 (= 28.9%) female authors, 60,097 (= 8.7%) unisex authors and 119,304 (= 17.1%) undefined authors were identified with a small inter-annual variability, as illustrated by S2 Fig. Unisex and undefined authors and their associated authorships (in total 532,784) were ignored in further analysis. In total, N = 1,469,925 authorships (*Life Science*: 600,386; *Chemistry*: 462,428, *Multidisciplinary*: 353,003 and *Earth & Environmental*: 73,172) form the basis for the analysis. The research output of a country was measured by considering the authorships of the affiliated institutions [16]. A single author is thus able to contribute with various authorships to the research output of different countries. Please note that the detection ratio essentially depends on the authors' country as illustrated by S3B Fig. Therefore, an adaptive threshold criterion θ for the exclusion of a country from the country-specific subanalysis was defined (S3A Fig), as recently described in Bendels et al. [14, 16]. In the present analysis, only countries with a detection ratio of at least 76.3% male and female authorships were included in the country-specific subanalysis. In particular, among the top 20 most productive countries, the Asian countries China, South Korea, Singapore, Taiwan (all with high rates of unisex names) and India (with many undefined names) were excluded. It is important to note that the threshold criterion was exclusively applied to the *country-specific* analysis. A general bibliometric overview is given in S1 Fig.

### Proportion of female authorships (FAP) & female authorship odds ratio (FAOR)

The analysis covers first-, co- and last-authorships. In our terminology, co-authorships comprise all authorships between *one* first and *one* last-authorship [7, 16]. Corresponding authors as well as equally distributed authorships were not considered. The proportion of female authorships (**FAP**) is defined as the quotient between the number of female authorships and the total sum of male and female authorships. For a better readability, the FAP is presented as percentage in the text. In addition, the female-to-male authorship odds ratio (**FAOR**) was calculated including the corresponding confidence intervals at a confidence level of 95%. The FAOR for e.g. the first authorship is calculated by FAOR_First_ = FemaleOdds_First_ / MaleOdds_First_, with FemaleOdds_First_ = FemaleN_First_ / (FemaleN_Co_+FemaleN_Last_) and MaleOdds_First_ = MaleN_First_ / (MaleN_Co_+MaleN_Last_) and FemaleN_t_ and MaleN_T_ defining the number of female and male, respectively, authorships of type t. The FAOR for first authorships is computed by considering all articles. By contrast, the FAORs for last and co-authorships are determined by considering all articles with at least two or three, respectively, authorships. Therefore, single authorships are considered as first authorships. For a concise notation, a triplet was used to indicate the sign of the *significant* female odd excess to secure a particular authorship. For example, the FAOR-triplet (+, =, -) indicates that women have a significantly higher odds ratio for first authorships, a non-significantly different odds ratio for co-authorships and a significantly lower odds ratio for last authorships compared to men. To increase the statistical significance, the FAP/FAOR-classification was applied for subjects (e.g. continents) with at least 1000 gender-identified authorships.

### Prestige Index

The *Prestige Index* is an indicator for the female odds excess of holding prestigious authorships compared to men [7, 16]. It is defined as the prestige-weighted average of the FAOR excess ε_t_ that is calculated over all authorship types t (i.e. for first, co- and last authorships), ε_t_ = w_t_ (FAOR_t_− 1), if FAOR_t_ ≥ 1, otherwise ε_t_ = w_t_ (1–1/FAOR_t_) with the weighting factor w_t_ [16]. In the examined journals and research areas, the prestige of authorships follows, by convention, a ranked order with a higher reputation of first and last authorships and a lower reputation of co-authorships [8]. Furthermore, we performed a test to exclude an alphabetic ordering of the author list (S5 Fig)[17]. Co-authorships were weighted negatively (w_co_ = –1), whereas first and last authorships were weighted positively (w_first_ = w_last_ = 1). This means higher odds for middle authorships decrease the *Prestige Index* whereas higher odds for first or last authorships increase the *Prestige Index*. A value of 0 indicates to a gender-neutral distribution of prestigious authorships, whereas a value above (below) 0 indicates to an excess (lack) of prestigious authorships held by women.

### Analysis of data

Average annual growth rates (AAGR) were employed to characterize the annual growth of the parameters. In order to assess the global future trend in female authorships a linear projection of FAP, FAOR, and *Prestige Index* has been carried out. To give a linear forecast for the year y > 2015, we first assessed the number of authorships N_t_ of type t by N_t_(y) = N_t_(2015) * (1 + AAGR_N_t_)^y-2015^, with AAGR_N_t_ defining the average annual growth rate of N_t_. Accordingly, the prediction of the FAP_t_ was calculated by FAP_t_(y) = FAP_t_(2015) * (1 + AAGR_FAP_t_)^y-2015^. The number of female and male authorships was then estimated by FemaleN_t_(y) = N_t_(y) * FAP_t_(y) and MaleN_t_(y) = N_t_(y) * (1—FAP_t_(y)), respectively, and applied to calculate the linear projection of FAOR and *Prestige Index*. The linear correlation between FAP, *Prestige Index* and Journal-5-Year-Impact-Factor was measured by the Pearson correlation. The statistical significance of differences between two FAPs was tested by a two-sample t-test. Kruskal-Wallis and post-hoc multi-comparison tests were applied to test the null hypothesis whether the not normally distributed (S4 Fig) citation rates were drawn from the same distribution. The significance threshold was set at .05. The analysis and illustration of data was performed using the MATLAB software (Natick, MA, USA).

## Results

### The global level

**Fig 1:**
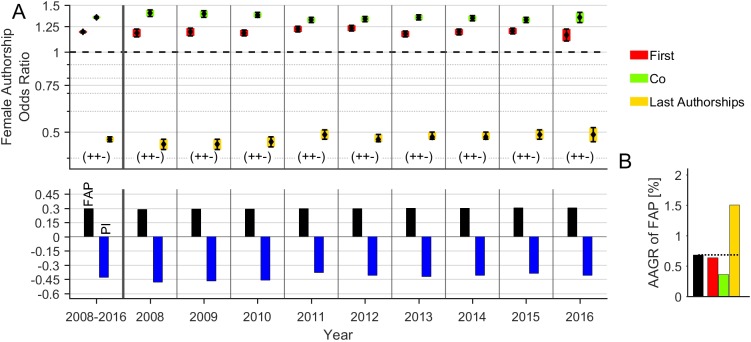
Time trend of female authorships on the global level. (**A**) The relative frequency of female authorships (FAP, bottom), the pattern of FAORs (with FAOR-triplet, top) and its associated *Prestige Index* (PI) are depicted by year and averaged over time. The very time-stable and unbalanced FAOR distribution is constantly characterized by the FAOR-pattern (+, +, -). The significantly negative PI points to a lack of prestigious authorships held by women. (**B**) The FAP exhibits a marginal increase as documented by its average annual growth rate (AAGR) of 0.7% per year with the highest rate for last authorships (1.5%).

On the global level we reveal an underrepresentation of female authorships with a FAP of 29.8%, relatively more female first (33.1%) and female co-authorships (31.8%) and a substantially less fraction of female last authorships (18.1%, Fig 1). The FAP shows a slight increase over the evaluation period (2008: 29.7%, 2016: 31.6%) with an AAGR of 0.7%. The highest AAGR was found for last authorships (1.4%), followed by first authorships (0.7%), and co-authorships (0.4%). The corresponding FAOR is 1.19 (CI: 1.18–1.20) for first authorships, 1.35 (CI: 1.34–1.36) for co-authorships and 0.47 (CI: 0.46–0.48) for last authorships. Thus, men have a more than 2-fold higher odds to secure last authorships on the global level. The differences are statistically significant (p<0.05) for all authorship types. As a result, the global pattern of FAORs is characterized by the FAOR-tuple (+, +, -), which is constantly present over the whole evaluation period. The associated *Prestige Index* is on average -0.42 indicating a lack of prestigious authorships held by women. The *Prestige Index* shows a tendency to increase in the last years (2008: -0.54, 2016: -0.41).

### Differences across continents

At the level of continents, the FAP ranges from 36.4% in South America to 19.8% in Asia (Table 1). The FAOR-tuple (+, +, -) constitutes the prevalent FAOR-pattern in all continents. In all continents, we found a negative *Prestige Index* ranging from -0.32 in South America to -0.76 in Asia.

**Table 1:** Female authorships by continents. The continents were descendingly ordered by the *Prestige Index*.

Continent Name	*Prestige Index*	FAP	FAOR Triplet	#Articles	#Authorships
South America	-0.32	36.4%	(+, +, -)	4982	11029
Australia & Oceania	-0.34	31.1%	(+, +, -)	11605	34328
North America	-0.39	29.6%	(+, +, -)	166677	658709
Europe	-0.49	32.5%	(+, +, -)	166245	556836
Asia	-0.76	19.8%	(+, +, -)	32515	124040

### Differences across countries

At the level of countries, we found a wide range of FAPs extending from 17.0% in Japan to 49.5% in Portugal (Table 2). Different FAOR-patterns were identified ranging from unfavorable with the FAOR-tuple (=, +, -) in Poland, Turkey, Argentina, Brazil, Norway, Russia, Czech Republic and Japan, to favorable with the FAOR-tuple (+, =, =) in New Zealand. The predominant FAOR-pattern is characterized by the FAOR-tuple (+, +, -), which is present in Italy, Spain, Israel, France, Greece, Belgium, Sweden, Australia, U.K., Ireland, Canada, the U.S., Austria, the Netherlands, Germany, Switzerland and Denmark.

**Table 2:** Classification of countries descendingly ordered by the Prestige Index.

Country Name	*Prestige Index*	FAP	FAOR Triplet	#Articles	#Authorships
Iceland	0.43	45.0%	(-, -, +)	375	1,743
New Zealand	0.07	29.0%	(+, =, =)	1,592	3,276
Finland	-0.11	39.6%	(+, =, -)	2,479	7,847
Portugal	-0.15	49.5%	(+, =, -)	1,503	3,328
Chile	-0.18	30.8%	(=, =, -)	686	1,342
Mexico	-0.21	36.6%	(=, =, -)	926	2,063
France	-0.29	36.7%	(+, +, -)	21,116	75,484
Spain	-0.32	40.4%	(+, +, -)	10,810	35,289
Argentina	-0.32	38.3%	(=, +, -)	988	2,359
Denmark	-0.33	26.1%	(+, +, -)	4,040	11,243
Norway	-0.37	32.6%	(=, +, -)	2,130	4,590
United States	-0.37	29.5%	(+, +, -)	150,166	609,233
Australia	-0.41	31.4%	(+, +, -)	10,007	31,040
Italy	-0.45	47.6%	(+, +, -)	9,928	36,123
Brazil	-0.46	37.7%	(=, +, -)	1,844	4,434
Sweden	-0.47	31.6%	(+, +, -)	6,312	17,847
United Kingdom	-0.49	31.0%	(+, +, -)	33,452	122,399
Ireland	-0.5	30.6%	(+, +, -)	1,689	4,244
Switzerland	-0.53	26.8%	(+, +, -)	11,100	31,921
Czech Republic	-0.56	30.3%	(=, +, -)	1,426	3,343
Hungary	-0.57	27.5%	(+, =, -)	979	2,199
Poland	-0.59	41.1%	(=, +, -)	1,703	3,717
Russian Federation	-0.59	32.1%	(=, +, -)	2,393	4,929
Canada	-0.59	30.0%	(+, +, -)	16,511	49,476
Austria	-0.61	29.2%	(+, +, -)	3,283	10,380
Belgium	-0.62	32.6%	(+, +, -)	4,083	12,945
Germany	-0.63	27.4%	(+, +, -)	35,045	130,957
Israel	-0.72	37.3%	(+, +, -)	4,640	14,354
Netherlands	-0.72	28.3%	(+, +, -)	9,085	30,151
Japan	-0.89	17.0%	(=, +, -)	26,190	106,830
Greece	-0.96	34.9%	(+, +, -)	1,036	2,144
Turkey	-2.15	40.3%	(=, +, -)	713	1,487

Interestingly, in almost all countries, women have significantly lower odds for last authorships compared to men. By contrast, Iceland is the only country, where women have significantly higher odds to secure last authorships. Its favorable FAOR with the FAOR-triplet (-, -, +) leads to the highest *Prestige Index* of all countries (0.43), followed by New Zealand (0.07) providing almost gender-neutral authorship odds (FAOR-triplet (+, =, =)). In all other countries, we revealed negative *Prestige Indices* pointing to a lack of prestigious authorships held by women (Table 2). In particular, in Turkey–with a high FAP of 40.3% and the most negative *Prestige Index* of -2.15—men have almost 6-fold higher odds for last authorships, whereas their female counterparts have more than 2-fold higher odds of a less prestigious co-authorship. We reveal no significant correlation between the FAP of a country and its corresponding *Prestige Index* (r(30) = .17, P>.05).

### Differences across journal categories

**Fig 2:**
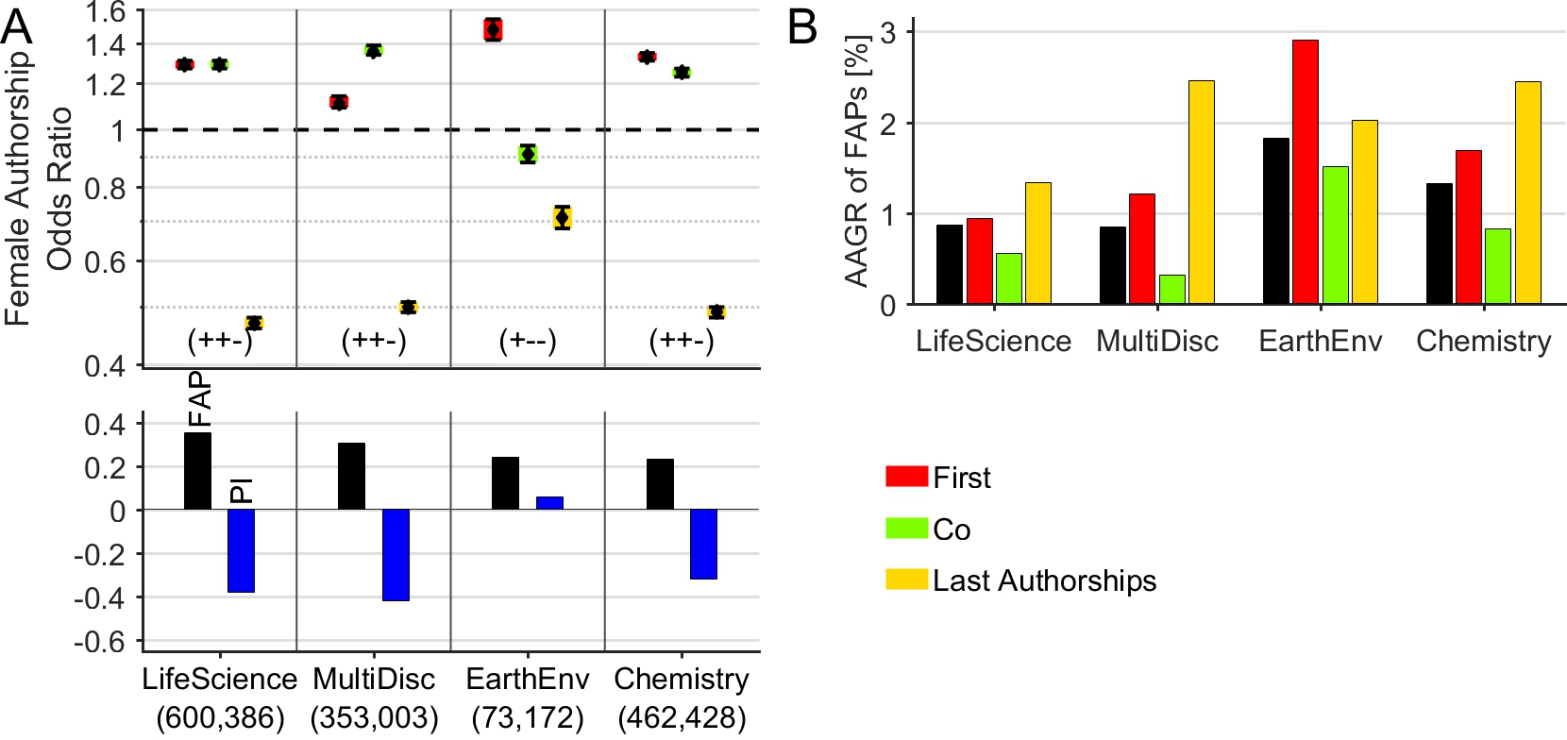
Female authorships by journal category. **(A)** The subject category *Earth & Environmental* has much favorable authorship odds for women than other categories. The number of considered male and female authorships is given in brackets. (B) In all categories, the FAP exhibits a positive annual growth with a relatively higher growth for first and last authorships and a relatively lower growth for co-authorships.

At the level of journal categories, we reveal the following FAPs: 35.3% for *Life Science*, 30.6% for *Multidisciplinary*, 24.0% for *Earth & Environmental*, and 23.2% for *Chemistry* (Fig 2A). Remarkably, in all categories, the FAP exhibits a positive annual growth with a relatively higher growth for first and last authorships and a relatively lower growth for co-authorships (Fig 2B). Specifically, the category-specific AAGRs of the FAP are 1.8% for *Earth & Environmental*, 1.3% for *Chemistry*, and 0.9% for both, *Life Science* and *Multidisciplinary*. The FAOR-patterns of *Life Science*, *Multidisciplinary* and *Chemistry* are characterized by the FAOR-tuple (+, +, -) with clearly negative *Prestige Indices*: -0.42 in *Multidisciplinary*, -0.38 in *Life Science*, and -0.32 in *Chemistry*. By contrast, the FAOR-pattern of the journal category *Earth & Environmental* exhibits the FAOR-tuple (+, -, -) with a positive *Prestige Index* of 0.06.

### Differences across journals

In a next step, we analyze the individual journals that were grouped by their category (Tables 3–6). For the category *Life Science*, we reveal FAPs that range from 26.0% in *Nature Methods* to 43.7% in the *American Journal of Human Genetics* (Table 3). Different FAOR-patterns are present: The most unfavorable pattern—characterized by the FAOR-tuple (-, +, -)—is found in *Nature Biotechnology*, by contrast, the journal *Ecology* has the most favorable FAOR-pattern with the tuple (+, =, -). In *all* journals of this category, men have significant higher odds for a last authorship. Furthermore, the consistently negative *Prestige Index*–ranging from -0.08 in *Ecology* to -1.04 in *Nature Biotechnology*—indicates that men have higher odds to secure prestigious authorships.

**Table 3:** Classification of *Life science* journals.

Journal Name	*Prestige Index*	FAP	FAOR Tuple	#Article	#Authorships
Ecology	-0.08	30.7%	(+, =, -)	2,554	8,846
Journal of Cell Biology	-0.20	37.7%	(+, +, -)	2,332	13,283
Cell Stem Cell	-0.20	35.3%	(+, +, -)	756	6,358
Proceedings of The Royal Society B-Biological Sciences	-0.20	30.7%	(+, +, -)	4,060	15,106
American Journal of Human Genetics	-0.23	43.7%	(+, +, -)	1,518	24,823
Developmental Cell	-0.24	38.5%	(+, +, -)	1,406	8,687
Journal of Biological *Chemistry*	-0.25	35.9%	(+, +, -)	28,960	151,659
Ecology Letters	-0.27	27.0%	(+, +, -)	875	4,071
Genes & Development	-0.31	35.9%	(+, +, -)	1,872	10,451
Journal of Neuroscience	-0.31	35.3%	(+, +, -)	12,971	63,737
Cancer Cell	-0.34	35.0%	(+, +, -)	830	10,239
Nature Structural & Molecular Biology	-0.36	31.7%	(+, +, -)	1,384	8,110
Molecular Cell	-0.37	34.6%	(+, +, -)	2,367	15,070
Embo Journal	-0.39	37.0%	(+, +, -)	2,164	14,539
Nature Cell Biology	-0.42	36.9%	(+, +, -)	1,098	7,951
Nature Genetics	-0.45	37.7%	(=, +, -)	1,678	49,132
Journal of Clinical Investigation	-0.45	36.8%	(+, +, -)	2,935	28,657
Cell Host & Microbe	-0.45	36.7%	(+, +, -)	785	6,348
Current Biology	-0.47	34.0%	(+, +, -)	2,953	15,094
Nature Neuroscience	-0.49	32.1%	(=, +, -)	1,700	10,795
Immunity	-0.52	37.2%	(+, +, -)	1,173	10,187
Plos Biology	-0.52	34.3%	(=, +, -)	1,598	10,592
Genome Research	-0.52	31.3%	(=, +, -)	1,625	13,173
Nature Immunology	-0.57	36.6%	(=, +, -)	952	7,927
Cell	-0.59	32.1%	(=, +, -)	2,858	24,710
Neuron	-0.63	31.3%	(=, +, -)	2,399	14,252
Nature Chemical Biology	-0.63	29.2%	(=, +, -)	947	6,804
Cell Metabolism	-0.72	34.2%	(+, +, -)	941	9,174
Nature Medicine	-0.76	35.0%	(=, +, -)	1,239	14,108
Nature Methods	-0.79	26.0%	(=, +, -)	1,133	8,306
Nature Biotechnology	-1.04	28.6%	(-, +, -)	817	7,775

**Table 4:** Classification of *Multidisciplinary* journals.

Journal Name	*Prestige* *Index*	FAP	FAOR Tuple	#Article	#Authorships
PNAS	-0.33	31.7%	(+, +, -)	29,742	175,332
Nature Communications	-0.47	30.2%	(=, +, -)	9,898	63,241
Science	-0.55	28.0%	(-, +, -)	6,567	46,264
Nature	-0.86	30.0%	(-, +, -)	6,907	68,924

**Table 5:** Classification of *Earth & Environmental* journals.

Journal Name	*Prestige Index*	FAP	FAOR-Tuple	#Article	# Authorships
Journal of Geophysical Research-Solid Earth	0.19	22.3%	(+, -, -)	3,389	7,855
Earth And Planetary Science Letters	0.12	23.4%	(+, -, -)	4,593	14,213
Journal of Geophysical Research-Atmospheres	0.12	22.7%	(+, -, -)	6,798	17,351
Journal of Geophysical Research-Oceans	0.05	23.1%	(+, =, -)	3,526	8,243
Geology	0.01	22.2%	(+, =, -)	2,323	7,890
Nature Geoscience	-0.07	22.1%	(+, =, -)	1,172	5,024
Ecology	-0.08	30.7%	(+, =, -)	2,554	8,846
Ecology Letters	-0.27	27.0%	(+, +, -)	875	4,071

**Table 6:** Classification of *Chemistry*-journals.

Journal Name	*Prestige Index*	FAP	FAOR Tuple	#Article	#Authorships
Nano Letters	-0.08	19.6%	(+, +, -)	8,300	33,049
Advanced Materials	-0.14	20.6%	(+, +, -)	6,620	24,082
Journal of Physical *Chemistry* Letters	-0.17	21.4%	(+, +, -)	4,143	14,373
Inorganic Chemistry	-0.18	25.7%	(+, +, -)	12,146	48,652
Analytical Chemistry	-0.25	27.2%	(+, +, -)	12,043	41,880
Nature Materials	-0.30	18.8%	(=, +, -)	1,157	5,380
Chemical Communications	-0.32	24.6%	(+, +, -)	24,274	69,384
Journal of The American Chemical Society	-0.36	22.1%	(+, +, -)	24,319	97,083
Chemical Science	-0.40	24.3%	(+, +, -)	3,202	12,367
Nature Nanotechnology	-0.48	20.8%	(=, +, -)	1,001	4,338
Organic Letters	-0.52	22.3%	(+, +, -)	12,891	36,493
Angewandte Chemie-International Edition	-0.54	22.2%	(+, +, -)	16,795	64,169
Nature Chemical Biology	-0.63	29.2%	(=, +, -)	947	6,804
Nature *Chemistry*	-0.80	21.1%	(=, +, -)	871	4,374

The FAP in *Multidisciplinary* journals ranges from 28.0% in *Science* to 31.7% in *PNAS* (Table 4). *Science* and *Nature* exhibit the most unfavorable FAOR-pattern, which is characterized by the tuple (-, +, -). In *all* journals of this category, men have significant higher odds for last authorships, whereas women have always-higher odds for co-authorships. The consistently negative *Prestige Index* ranges from -0.33 in *PNAS* to -0.86 in *Nature*.

The FAP for *Earth & Environmental* journals ranges from 22.1% in *Nature Geoscience* to 30.7% in *Ecology* (Table 5). In all journals of this category, women have higher odds for a first authorship, whereas men have higher odds for a last authorship. Best female odds to secure prestigious authorships are given in the three editions of the *Journal of Geophysical Research* (*Prestige Index* ranges from 0.05 to 0.19) and *Earth and Planetary Science Letters* (*0*.*12*). Best male odds for prestigious authorships are found in *Ecology Letters* (*Prestige Index* = -0.27).

The last category, *Chemistry* journals, is characterized by relatively low FAPs ranging from 18.8% in *Nature Materials* to 29.2% in *Nature Chemical Biology* (Table 6). In all *Chemistry* journals, men have significant higher odds for last authorships, whereas women have higher odds for co-authorships, as described above for *Multidisciplinary* journals. The *Prestige Index* is consistently negative ranging from -0.80 in *Nature Chemistry* to -0.08 in *Nano Letters*.

In summary, in only 5 out of 54 journals women have equal or higher odds for prestigious authorships compared to men (*Earth & Planetary Science Letters*, the *Journals of Geophysical Research*, and *Geology*).

### Correlation of journal parameters

**Fig 3:**
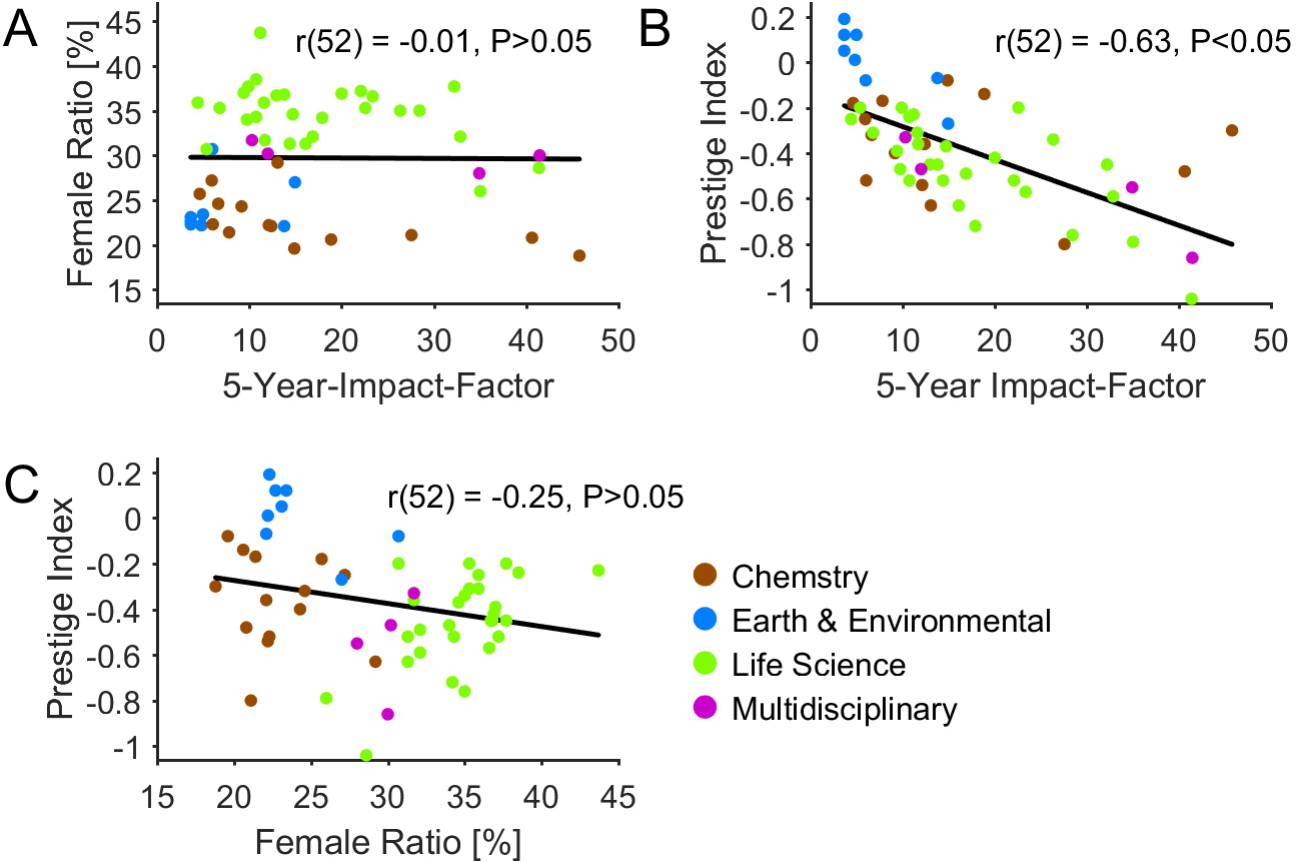
Correlation of journal parameters. (A) There is no correlation between the 5-Year-Impact-Factor of a journal and its FAP. (B) A large negative correlation between the 5-Year-Impact-Factor of a journal and its *Prestige Index* was revealed. (C) The graph shows a small, but not significant negative correlation between the FAP and the *Prestige Index* of a journal.

We reveal a large negative correlation between the 5-year-impact-factor of a journal and its corresponding *Prestige Index* (r(52) = -.63, p < .001, Fig 3B). This means, the higher the 5-year-impact factor of a journal is, the lower are the female odds to secure prestigious authorships. By contrast, we do not reveal a correlation between FAP and 5-Year-Impact-Factor of a journal (r(52) = -.01, P>.05, Fig 3A) and between the FAP of a journal and its *Prestige Index* (r(52) = -.25, P>.05, Fig 3C). However, there is a tendency that a high FAP in a journal is associated with a disproportional high ratio of female co-authorships (Fig 3C).

### Female authorships by authors per article

**Fig 4:**
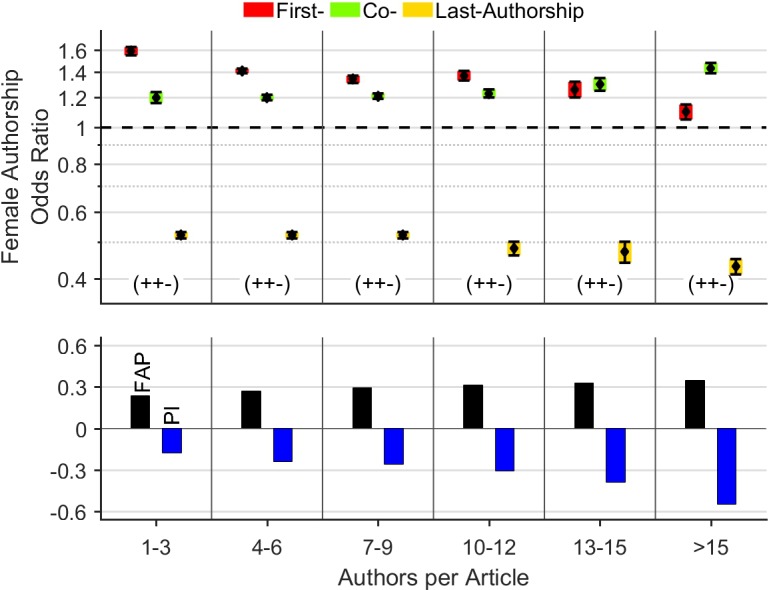
Female authorships by authors per article. The more authors contribute to an article, the higher is the FAP and the lower is the representation of women at prestigious authorships.

We also inspect the role women tend to have in collaborative projects, indicated by the number of authors per article. We found a statistically significant (t(25162), p < .001) increase of the FAP from 23.5% for articles with 1–3 authors to 34.9% for articles with more than 15 authors (Fig 4). Concomitantly, the FAOR for prestigious first or last authorships decreases (first: 1.59 to 1.1, last: 0.52 to 0.43), whereas the FAOR for less prestigious co-authorships increases (1.2 to 1.43); the differences were statistically significant. Overall, this leads to a continuous and statistically significant decrease of the *Prestige Index* from -0.18 for articles with 1–3 authors, to -0.55 for articles with more than 15 authors. In statistical terms, the more authors contribute to an article, the higher is the FAP and the lower is the representation of women at prestigious authorships.

### Citation & productivity analysis

**Fig 5:**
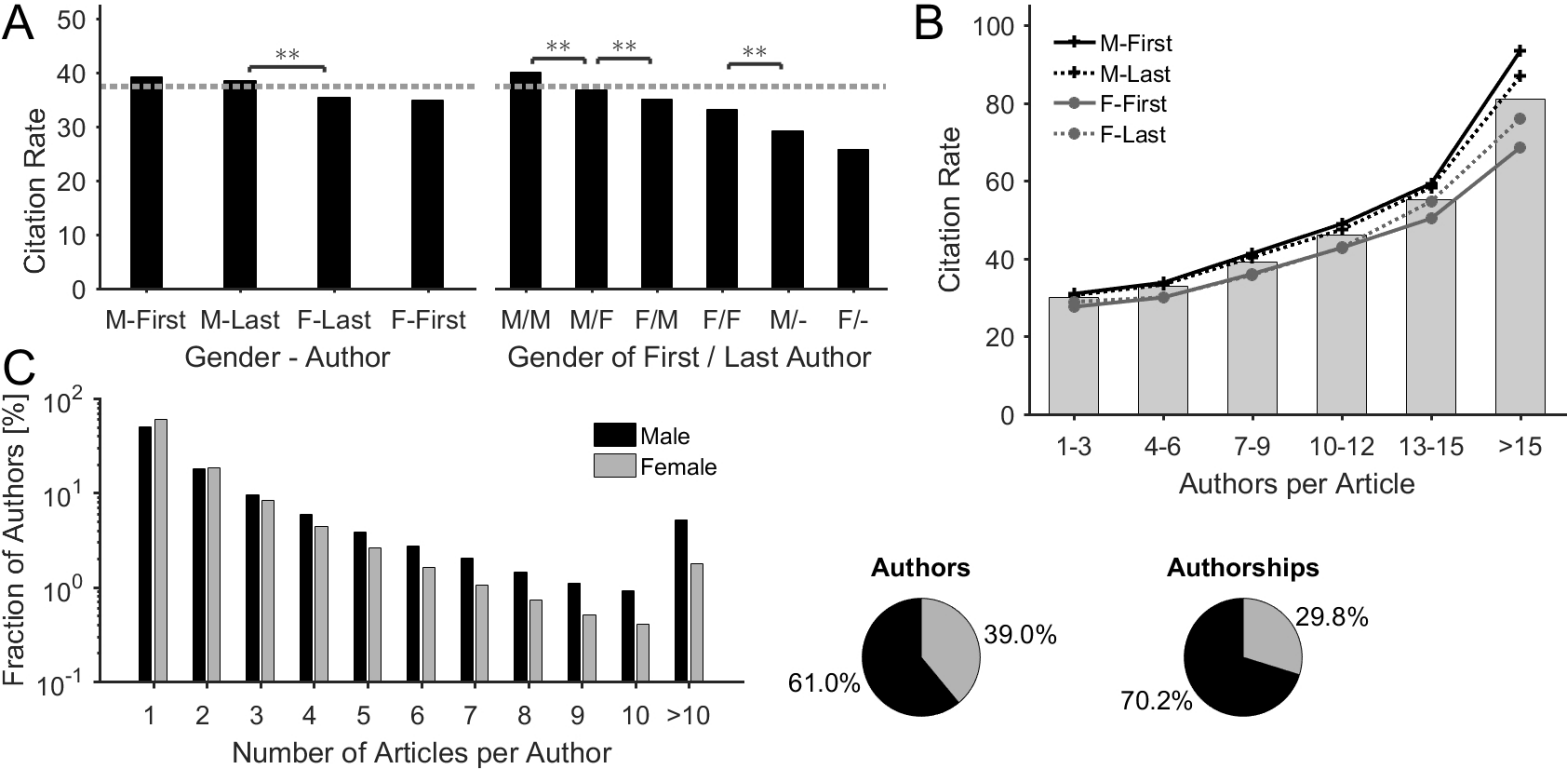
Gender-specificity of citations & scholarly productivity. (**A**) The descendingly ordered citation rates shows that articles with male key authorships are more frequently cited than articles with female key authorships. The mean citation rate of 37.5 citations/article is depicted by a dotted line (Kruskal-Wallis test, (*): p < .05 (**): p < .01). (**B**) Average citation rates of both, ungrouped articles (bars) and articles that were grouped by the gender of their key authorships (lines), plotted as a function of the number of authors. Statistically, the citation rate of an article is higher the more authors are involved. The differences in citation rates between the two genders increase with the number of authors per article. (**C**) Gender-specific distribution of the number of articles per author. Women dominate the sub-groups 'author has 1 or 2 article(s)'. All other sub-groups are characterized by a relatively over-representation of male authors. This finding correlates with the higher productivity of male authors, as 61.0% male authors are responsible for 70.2% of all authorships.

The citation analysis reveals that articles with male key authors are more frequently cited than articles with female key authors (Fig 5A). Specifically, articles with a male first or last author have citation rates of 39.2 and 38.6 citations/article, respectively, whereas articles with a female last or first author exhibit citation rates of 35.4 and 34.9 citations/article, respectively. The differences are statistically significant (p<0.01) between male and female groups, but not within a gender group. Articles with a female key authorship are on average below the mean citation rate of 37.5 citations/article. The analysis of combined authorships documents that male-first/male-last and male-first/female-last articles have on average the highest citation rates with 40.2 and 36.8 citations/article, respectively, followed by female-first/male-last and female-first/female-last articles with 35.2 and 33.2 citations/article, respectively (Fig 5A, right). Single-authored articles with a female author have the lowest citation rates with 25.9 citations/article, which differ not statistically from those with a male author (29.2 citations/article). Statistically, the citation rate of an article becomes higher the more authors are involved (Fig 5B), as e.g. the average citation rate of articles with 1–3 authors is 30.0, whereas articles with more than 15 authors are cited on average 80.9 times. The differences in the citation rates between articles with male or female key authorships impose at each article's author count level and increase the more authors contribute to an article (Fig 5B).

Regarding scientific productivity, the analysis reveals marked differences between the two genders: Women clearly dominate the sub-groups 'author has one article' and 'authors has two articles', as e.g. 60.0% of the female authors, but only 49.5% of the male authors had published a single article in our dataset (Fig 5C). By contrast, all other sub-groups—with authors that published more than two articles—are characterized by an over-representation of male authors, which becomes higher with increasing productivity. Particularly the sub-group of most productive authors is clearly dominated by men, as 5.2% of the male authors but only 1.8% of the female authors published more than 10 articles. Overall, 61.0% male authors are responsible for 70.2% of all authorships in our data set (Fig 5C), thus indicating a higher productivity of the male authors.

## Discussion

### Underrepresented at leading positions

In this descriptive study, we analyze the representation of female authorships for the field of high-quality research covering the areas *Life Science*, *Earth & Environmental*, *Multidisciplinary* and *Chemistry*: The global FAP of 29.0% corresponds very precisely to the previous determined proportion of female authorships for the whole area of science (30%) as published by Lariviere et al. in 2013 [6], but is significantly lower than the FAP revealed for six high-impact medical journals (34.0%) [12] and the research fields of dermatology (43.0%), epilepsy (39.4%) [16], schizophrenia (37.6%) [7], and stroke medicine (36.3%). The proportion of female first (33.1%) and last authorships (18.1%) is higher than the percentage of 29.3% and 14.5%, respectively, that was found for five high-impact gastroenterology journals in 2012 [4]. We identified a global pattern of FAORs that is characterized by the triplet (+, +, -), i.e. higher female odds for first or co-authorships and lower female odds for last authorships compared to men. This uneven distribution of female authors across the different authorships reflects the known structural imbalance of the scientific system [4, 7, 18–21], with just a few female group leaders as last-authors and many female researchers at lower hierarchical level being first- or co-authors.

Moreover, the FAOR-distribution documents a significant lack of prestigious authorships held by women. Numerically, this result stems from the fact that the high FAOR for first-authorships does not compensate the unfavorable FAORs for co- or last authorships. This finding is remarkable, since academic publishing at high prestigious authorships is the core element of career advancement in science [7, 22–24]. Reasons for the relative overrepresentation of female co-authorships could be that the female contributions to articles are less significant than their male coauthors, thereby shifting to lower prestige co-author positions, as discussed by West et al. [3]. Another mechanism could be that men negotiate more successfully for the more prestigious authorship positions [3].

### Position affects productivity and citation rate

The lower productivity of female scientists in the field of high-impact research (39% female authors hold 28.9% of the authorships) is consistent with reports from other scientific fields [4, 6, 16, 18, 20, 21, 25]. In particular, we were able to reproduce the clear male overrepresentation at the highest levels of productivity, as shown by Symonds and colleagues [23] for the field of ecology and evolutionary biology. According to our experience, we think that the main factors affecting women's productivity are not higher rejection rates as e.g. demonstrated for the journals *Nature Neuroscience*[26] and *Cortex[27]*. It is more likely that the output and submission rates themselves differ by the particular rank of a scientist with a considerable higher output of the primarily male senior scientists [7, 21]. In addition, Reed and colleagues [25] were able to show that that publication rates of men start to increase earlier in their careers compared to women.

What about citations of publications with female key authors? Many previous studies across various disciplines reported that female authors attract fewer citations than their male counterparts [3, 6, 21, 23, 28, 29]. We here extend the results to high impact science and show, that multi-author articles with female authors in key positions are also less frequently cited than those with male key authors. A reason for this is surely the finding that women are less likely to secure prestigious authorships in articles with many authors (Fig 4) attracting the highest citation rates (Fig 5) [30]. This assumption is supported by the fact that single-author articles exhibit no statistically relevant gender disparity in citation rates (Fig 5A, right). This effect is aggravated by the finding that the differences in citation rates between the two genders *increase* with the number of authors per article (Fig 5B). This means that women are not only relatively more underrepresented at high-impact key authorships, but also attract significantly fewer citations for (high-impact) key authorships compared to the men. It is plausible to assume that the lack of women in leadership positions causes this accentuated female under-representation (*structural reasons)* [22] since the distribution of key authorships follows, by convention, a hierarchical order [8]. This is even more valid for the highly competitive key authorships in articles with many authors, e.g. collaborative articles. To conclude, the distribution of key authorships in high-impact collaboration articles emerges as one important factor in the generation of gender-specific citation rates. However, it remains unclear, why articles with female key authorships attract constantly fewer citations, particularly also for articles with few authors, as shown by Fig 5B. The reasons for this may be also found in structural reasons as the mainly male senior scientists tend to have more strongly scientific (citation) networks than the female early-career researches [31]. Moreover, men cite themselves more than women do, as shown by Chawla [32]. Methodically, it should be mentioned that the citation analysis mainly covers the situation from the early phase of investigation (2008–2010), due to the time-delayed occurrence of citations and the thus stronger impact of older articles ("Cited Half-Life") [33]. The relatively high average citation rate of 37.5 citations per article compared to other studies from a similar time interval [7, 28], reflects both, scientific quality and impact of the examined articles. It should also be mentioned, that number of citations is dependent on the scientific area.

### Most productive countries characterized by high degree of uniformity

When analysing individual countries, we also identify the FAOR-tuple (+, +, -) as being predominant and nearly exclusively present among the 15 most productive countries regardless of female authorship frequencies ranging from 26.1% in Denmark to 49.5% in Italy (Table 2). This surprisingly high uniformity in the gender-specific career dichotomy across the most productive countries is remarkable. However, it is also a sign for the high proportion of early-career female researchers in these countries, who have entered the academic field in the last decades [34, 35]. Japan–with a strong sense of patriarchy in society [36]—occupies a noticeable role among the top 15 productive countries: it has with 17% the lowest FAP, an even more unfavourable FAOR-pattern and a relatively low *Prestige Index*, thus pointing to a non-advanced integration of female scientists. Concomitantly, the Japanese government recently reported that its world standing in science and technology is falling [37] and introduced a range of policies in response to this, which are designed to recruit top international researchers [38].

By contrast, the countries New Zealand and Iceland may offer inspiration for improving female participation in scientific publishing: These are the only two among the 30 top publishing countries where women have more favorable odds to secure prestige authorships than men. This result strongly correlates to the Global Gender Gap Report 2016, in which Iceland and New Zealand were ranked 1^st^ and 9^th^, respectively, out of a total of 144 countries in the world[39]. Both countries have had a long history of promoting women's equality as e.g. New Zealand was the first country in the world to give women the right to vote in 1893 by the Electoral Act. Interestingly, Iceland was for centuries a seafaring nation where women were temporarily left to rely on themselves as their husbands traversed the oceans. Today almost 80% of Iceland's women work. Furthermore, as result of mandatory quotas, almost half of board members of listed companies are women, while 65% of Iceland's university students and 41% of the Member of Parliament are female[40]. The Economist recently named Iceland as the world’s best place for working women[41].

Methodically, the comparison of e.g. Turkey to Finland emphasizes the importance to include FAORs and the related *Prestige Index* in the analysis of authorships (Table 2): Although both countries are characterized by a relatively high proportion of approximately 40% female authorships, Finland has a more favourable FAOR-pattern with considerably higher female odds for prestigious authorships than Turkey. Apparently, the FAOR-distribution reveals two completely different scientific systems regarding the integration of women.

### Advanced integration of women in *Earth & Environmental*

The analysis of journal-categories confirm previous findings regarding the participation of women with highest percentages for the category *Life Science* and lowest percentages for the category *Chemistry*[6]. It remains unclear, why the subject category *Earth & Environmental* has so much favorable authorship odds for women as well as higher AAGRs of female authorships than other categories. However, this outcome is in line with the finding that after the step to first academic position, men and women are promoted from assistant to associate professor at PhD-granting U.S. institutions at comparable rates regardless of the low proportion of female scientists in this scientific field [42]. Moreover, it was recently reported that women have a higher acceptance rate in American Geophysical Union journals than men [43], thus documenting an well-advanced integration of female scientist in this scientific area.

### Accentuated female underrepresentation at top journals

At the level of individual journals, we reveal a striking uniformity with respect to female authorship odds: Specifically, in only 5 out of 54 journals women have equal or slightly better odds to secure prestigious authorships. Furthermore, in all journals men have higher odds to be a last-author. Finally, in only 3 out of 54 journals women have lower odds to get a co-authorship compared to men. Evidently, the global gender-imbalances in high impact research are consistently mapped to the related journals. Most remarkably, our analysis further reveals a negative correlation between the 5-Year-Impact-Factor of a journal and the female odds to secure prestigious authorships (Fig 3B). Evidently, the underrepresentation of women accentuates at highly competitive key authorships as shown here for top journals and for articles with many authors. In this context, a parallel can be drawn to studies reporting about significant gender differences in competitive attitudes [44, 45] and power-related goals [46].

### Results contradict the socio-dynamic theory of critical mass

Overall, our analysis clearly demonstrates that countries (like New Zealand), journal categories (like *Earth & Environmental* Journals) or individual journals (like the *Journal of Geophysical Research*) with a low proportion of female authorships can still provide favourable conditions for women to be driving forces in publishing of high-quality science. This finding contradicts the socio-dynamic theory of critical mass [47] stating that ‘with an increase in relative numbers, minority members are potentially allies, can form coalitions, and can affect the culture of the group’ [48]. Interestingly, we reveal a negative correlation between FAP and *Prestige index* when grouping articles by author count (Fig 4). This finding suggests that, globally, there is a tendency that high FAPs are regularly associated with a disproportionate high ratio of female co-authorships.

### Methodical limitations

This study documents how deriving information about the state of integration of women in the field of high impact science can be done by a bibliometric approach analyzing the gender-specific distribution of authorships. Conceptually, we extend frequency-based approaches [34, 49–51] by considering the female-to-male odds ratios of authorships as well as their different prestige that was measured by a straightforward weighting scheme. Depending on the question posed, other weighting schemes are permissible. The most important limitation related to this fully algorithmic approach with its minimized inter-individual variability is the lack of information regarding academic rank (e.g. Assistant vs. Associate Professor) and degree (e.g. master's degree vs. doctoral degree) of a scholar, its age and employment status (full time vs. part time), which can only be obtained by personal communication, questionnaires or manual inspection of e.g. online profiles, as demonstrated by other studies [4, 21, 34]. Another limitation of our approach is the fact that we had to exclude countries from the *country-specific* analysis due to a relative high fraction of unisex or unknown first names. The reason behind this is that it is not possible to assess reliably the amount of female or male authors behind a large fraction of e.g. unisex names determined in many Asian countries.

### Outlook

To summarize, previous efforts for improving gender equity in high-quality science were successful with respect to relative frequencies and early-career steps as shown here and elsewhere[3]. However, the results are multi-faceted since low female senior author odds were found in every system level screened (continents, countries, journal categories, journals, and authors per article).

**Fig 6:**
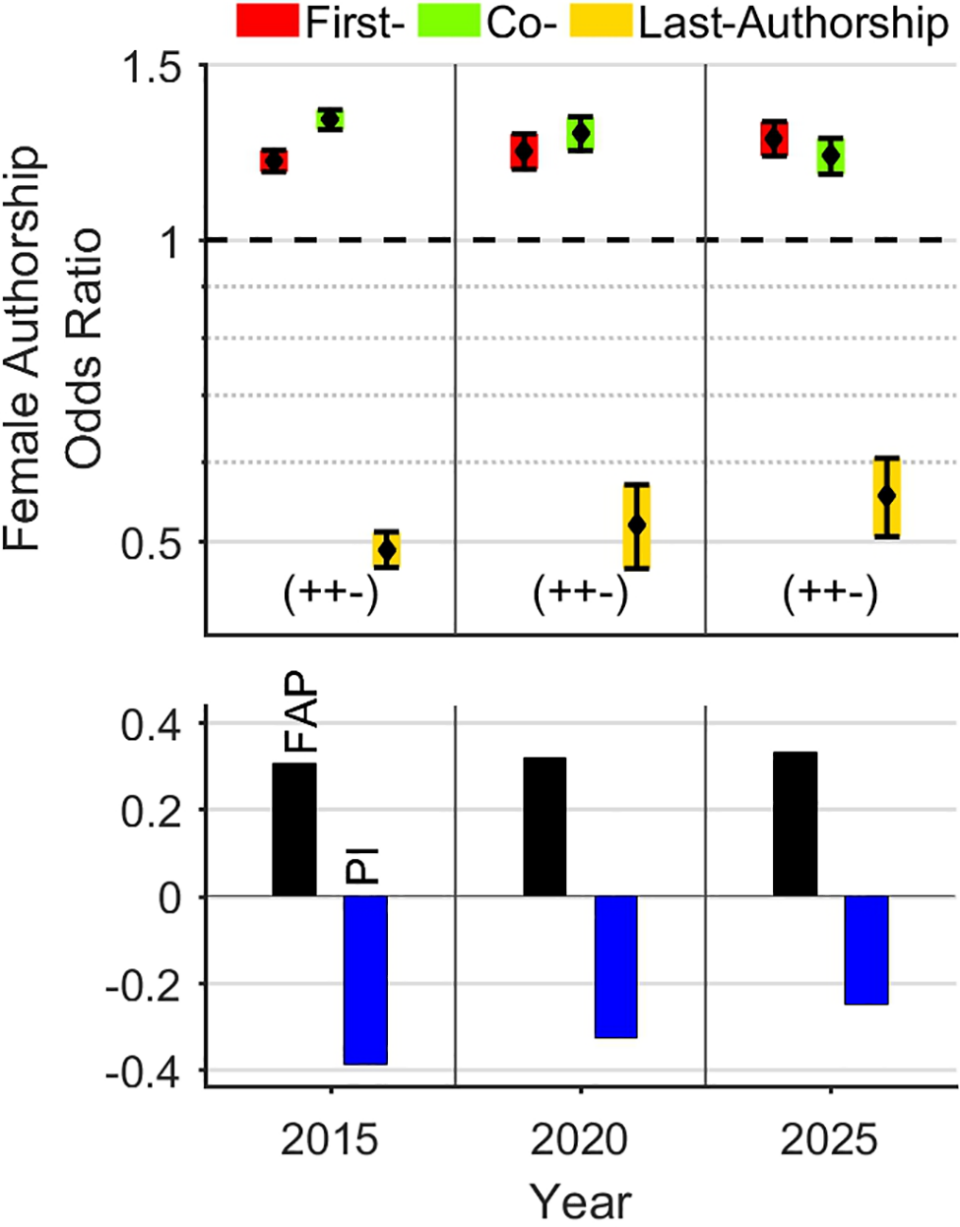
Linear projection of the development of female authorships on the global level. The prognosis for the next decades forecasts a slow harmonization of authorship odds between the two genders and the persistence of the unfavorable FAOR-pattern with a negative *Prestige Index*. A FAP of 33.1% is prognosticated for the year 2025.

Concerning this remarkable gender-specific career dichotomy, it can be argued that one should expect some lag between imbalances in the first and last author positions, as it takes time for younger scholars to become leaders of research groups [3, 4, 28]. This demographic shift seems plausible, as a continuous rise of female first-authorships in the last years has been described in several academic fields [4, 6, 21, 28]. However, we hypothesize in accord with West et al.[3], that this structural imbalance will not change significantly in near future in the field of high quality research due to low annual increase of the FAP (0.7% per year). Moreover, the described unfavorable FAOR-pattern and the negative *Prestige Index* were continuously present over the whole evaluation period (2008–2016) with a remarkable numerical stability. According to this estimation, a quantitative prognosis of the temporal development of female authorships on the global level up to the year 2025 documents only a particularly slow harmonization of authorship odds and the persistence of the unfavorable FAOR-pattern (Fig 6). In this prognosis, the *Prestige Index* will remain negative and the FAP is forecast to reach a value of 33.1% in the year 2025. Based on this consideration, we do not except any fundamental changes in the next decade regarding both female authorship odds and frequencies. In line with this assessment, various studies recently report about a remarkable persistence of the gender-specific career dichotomy despite a considerable increase in female first authorships [4, 7, 19, 20, 34].

### Conclusions

Given these findings, fundamental questions arise: Does the academic system has to redefine the esteem for female leadership? Will we require an outside-the-box thinking in academic institutions, editorial boards and funding agencies? With regard to recent studies, demonstrating significant gender differences in competitive attitudes [44, 45], power-related goals [46], life priorities [6], and career preferences [52]: will the gender gap in senior positions ever be closed completely?

Regardless which actions are planned to offer new perspectives for female academics, they should not solely focus on relative frequencies but rather strengthen strategies to improve female-specific (authorship) chances in order to overcome the career dichotomy between the two genders. Quantitative indicators could be used as a valuable instrument to monitor the future development. Specifically, the implementation of an annual gender-specific academic rating of institutions and countries may help to seal the persistently "leaky pipeline" of female scientists.

## Supporting information

S1 Fig Bibliometric overview. (A) The article count increases from 30,599 in 2008 to 38,276 in 2015; the average annual growth rate (AAGR) is 3.3%. (B) The number of authors per article (author-rate) increases from 5.92 authors/article in 2008 to 7.68 authors/article in 2016. (C) The percentage of international collaboration articles monotonically increases from 0.29 in 2008 to 0.40 in 2016 with an AAGR of 4.1%. (D) The fraction of articles grouped the gender of their key authors' documents a quantitative superiority of articles with male key authorships. (E) The fraction of articles is depicted by country (bar plot) and by continent (pie charts). Please note that the sum of percentages is greater than one due to international collaborations. AU = Australia, CA = Canada, CH = Switzerland, CN = China, DE = Germany, ES = Spain, FR = France, GB = United Kingdom, IN = India, IT = Italy, JP = Japan, KR = South Korea, NL = Netherlands, SE = Sweden, US = United States.
(JPG)

S2 Fig Gender detection output by time. The ratios of detected male, female, unisex and undefined authorships ordered by publication year document a relatively small inter-annual variability.
(TIF)

S3 Fig Quality of algorithmic gender detection by country. (**A**) An adaptive threshold country criterion θ for the inclusion of a country in the country-specific gender analysis was defined by a ROC-like curve incorporating both detection ratio and cumulative author count [14]. In this study, countries with a detection rate of at least θ = 0.763 male + female authors (i.e. 76.3% of all authorships) from N = 95 countries were included in the country-specific analysis. Countries with a large amount of authors are indicated by country code. (**B**) The result of the algorithmic gender detection—classified as male/female, unisex or undefined—grouped by countries that are ordered in descending order by their publication count, documents a relative high frequency of male/female authors for most of the top 20 countries, with the exception of the Asian countries China (CN), South Korea (KR), Singapore (SG), Taiwan (TW) and India (IN). The latter countries are characterized by a high frequency of unisex (CH, KR, TW, SG) or unknown names (IN) and are excluded (**X**) from analysis due to the threshold criterion θ (dotted line). AU = Australia, BE = Belgium, CA = Canada, CH = Switzerland, CN = China, DE = Germany, DK = Denmark, ES = Spain, FR = France, GB = United Kingdom, IL = Israel, IN = India, IT = Italy, JP = Japan, KR = South Korea, NL = Netherlands, SG = Singapore, SE = Sweden, TW = Taiwan, US = United States.
(TIF)

S4 Fig Probability density function of the citation rate. The semi-logarithmic plot of the citation count per article (= citation rate) exhibits an exponential-like decreasing probability density function with a mean citation rate of 37.5 citations/article.
(TIF)

S5 Fig Test for alphabetical ordering of the author list. The proportion of publications with an alphabetic ordered author list is depicted with respect to the authors per article (blue). The values correspond very closely to those obtained for randomly ordered author lists (yellow).
(JPG)

## Abbreviations

AAGR: Average Annual Growth Rate
FAOR: Female Authorship Odds Ratio
FAP: Proportion of Female Authorships
PI: Prestige Index
