# Supplementary figures and images for: Gender disparities in high-quality research revealed by Nature Index journals
Source: PLoS One. 2018 Jan 2;13(1):e0189136. doi: 10.1371/journal.pone.0189136 (PMC5749692; doi:10.1371/journal.pone.0189136)

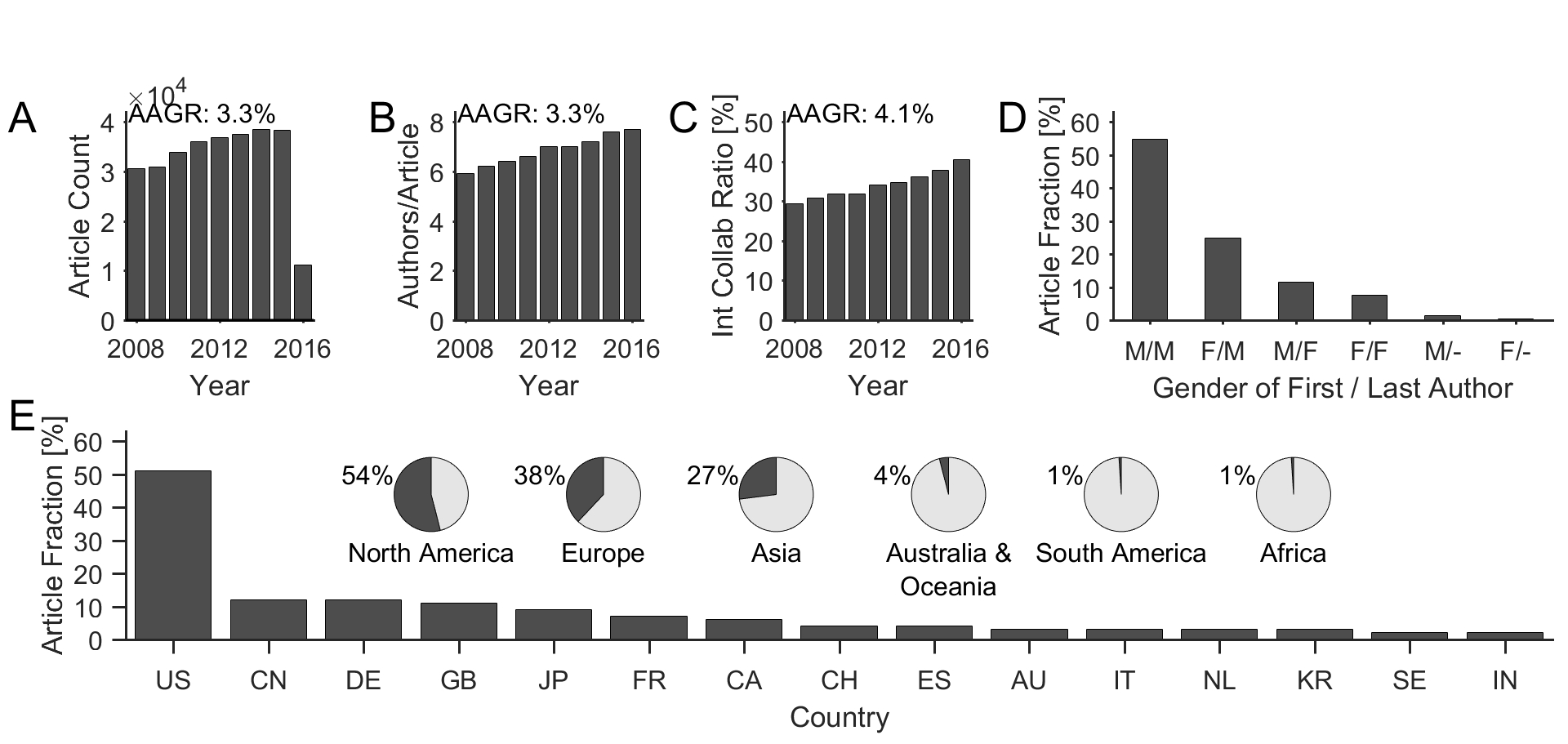

Supplement: S1 Fig — (A) The article count increases from 30,599 in 2008 to 38,276 in 2015; the average annual growth rate (AAGR) is 3.3%. (B) The number of authors per article (author-rate) increases from 5.92 authors/article in 2008 to 7.68 authors/article in 2016. (C) The percentage of international collaboration articles monotonically increases from 0.29 in 2008 to 0.40 in 2016 with an AAGR of 4.1%. (D) The fraction of articles grouped the gender of their key authors' documents a quantitative superiority of articles with male key authorships. (E) The fraction of articles is depicted by country (bar plot) and by continent (pie charts). Please note that the sum of percentages is greater than one due to international collaborations. AU = Australia, CA = Canada, CH = Switzerland, CN = China, DE = Germany, ES = Spain, FR = France, GB = United Kingdom, IN = India, IT = Italy, JP = Japan, KR = South Korea, NL = Netherlands, SE = Sweden, US = United States. (JPG) [file pone.0189136.s001.jpg]

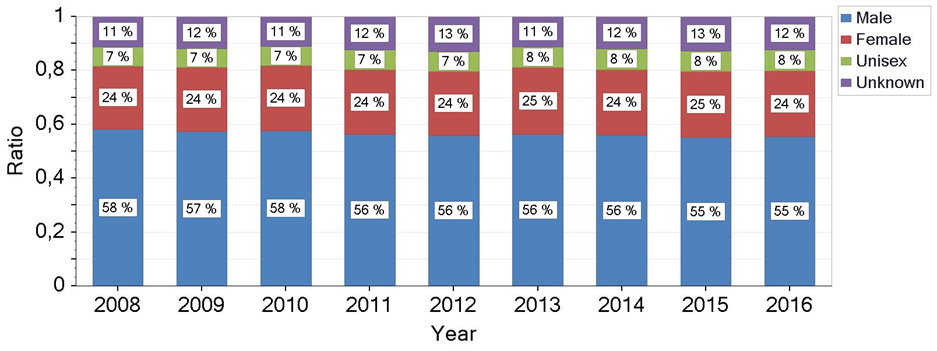

Supplement: S2 Fig — The ratios of detected male, female, unisex and undefined authorships ordered by publication year document a relatively small inter-annual variability. (TIF) [file pone.0189136.s002.tif]

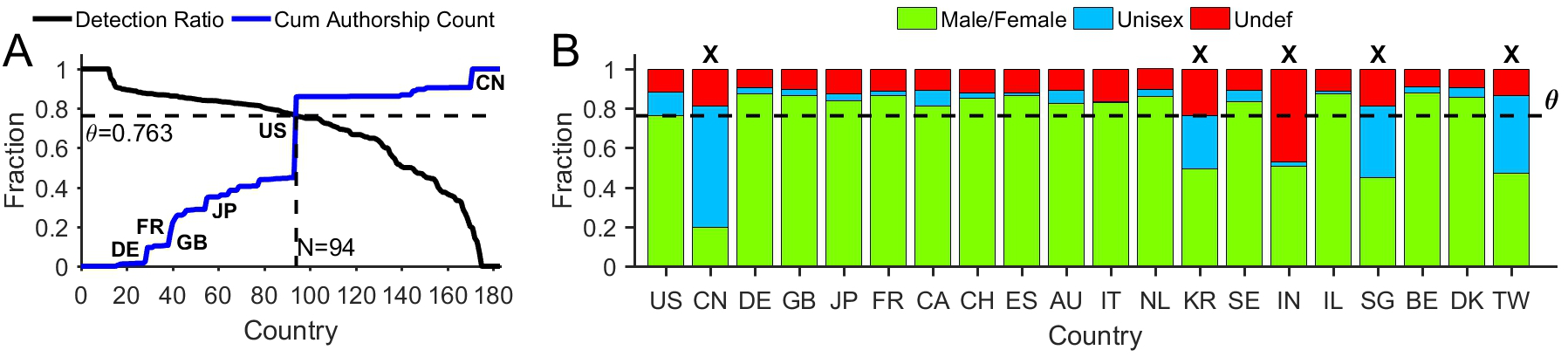

Supplement: S3 Fig — (A) An adaptive threshold country criterion θ for the inclusion of a country in the country-specific gender analysis was defined by a ROC-like curve incorporating both detection ratio and cumulative author count [14]. In this study, countries with a detection rate of at least θ = 0.763 male + female authors (i.e. 76.3% of all authorships) from N = 95 countries were included in the country-specific analysis. Countries with a large amount of authors are indicated by country code. (B) The result of the algorithmic gender detection—classified as male/female, unisex or undefined—grouped by countries that are ordered in descending order by their publication count, documents a relative high frequency of male/female authors for most of the top 20 countries, with the exception of the Asian countries China (CN), South Korea (KR), Singapore (SG), Taiwan (TW) and India (IN). The latter countries are characterized by a high frequency of unisex (CH, KR, TW, SG) or unknown names (IN) and are excluded (X) from analysis due to the threshold criterion θ (dotted line). AU = Australia, BE = Belgium, CA = Canada, CH = Switzerland, CN = China, DE = Germany, DK = Denmark, ES = Spain, FR = France, GB = United Kingdom, IL = Israel, IN = India, IT = Italy, JP = Japan, KR = South Korea, NL = Netherlands, SG = Singapore, SE = Sweden, TW = Taiwan, US = United States. (TIF) [file pone.0189136.s003.tif]

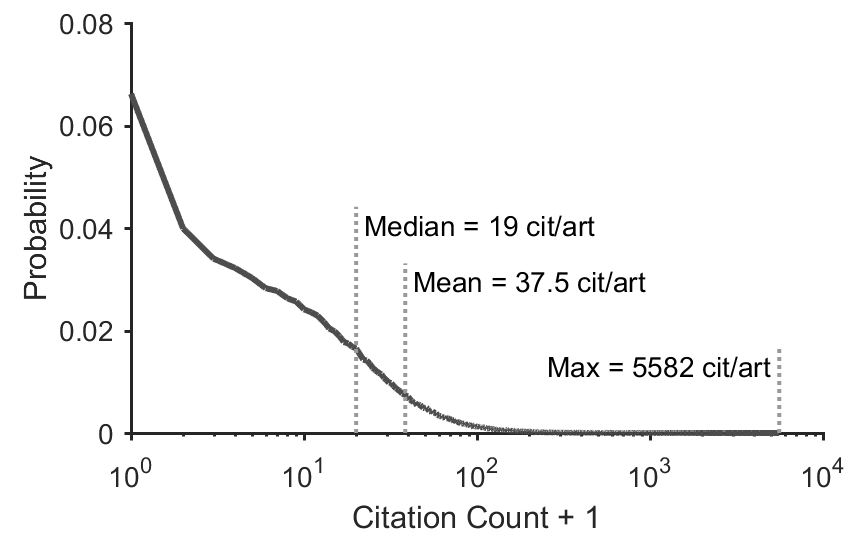

Supplement: S4 Fig — The semi-logarithmic plot of the citation count per article (= citation rate) exhibits an exponential-like decreasing probability density function with a mean citation rate of 37.5 citations/article. (TIF) [file pone.0189136.s004.tif]

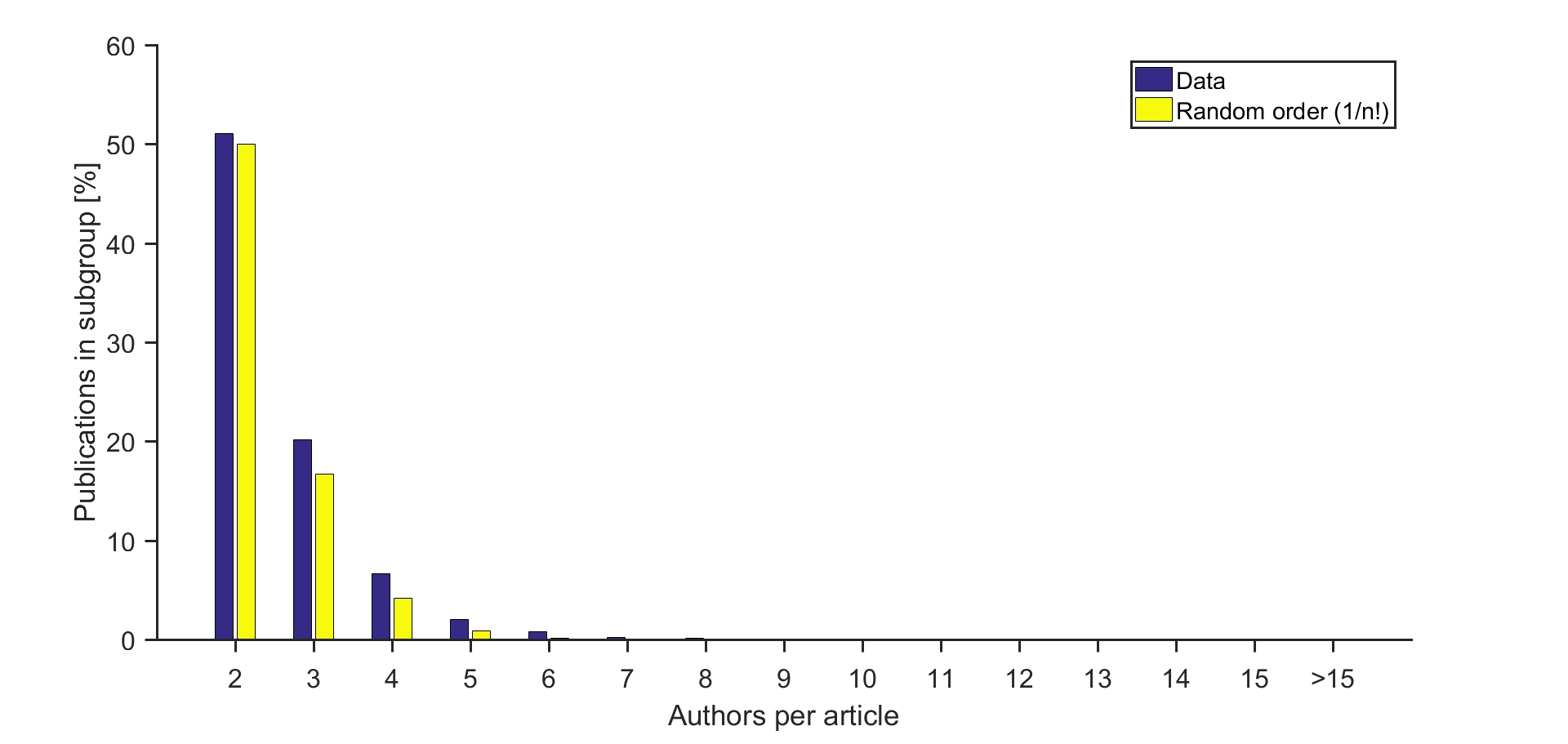

Supplement: S5 Fig — The proportion of publications with an alphabetic ordered author list is depicted with respect to the authors per article (blue). The values correspond very closely to those obtained for randomly ordered author lists (yellow). (JPG) [file pone.0189136.s005.jpg]
